# Evaluation of Liver Iron Content by Magnetic Resonance Imaging in Children with Acute Lymphoblastic Leukemia after Cessation of Treatment

**DOI:** 10.4274/tjh.galenos.2020.2019.0364

**Published:** 2020-11-19

**Authors:** Sezer Acar, Salih Gözmen, Selen Bayraktaroğlu, Sultan O. Acar, Neryal Tahta, Yeşim Aydınok, Raziye C. Vergin

**Affiliations:** 1 Dr. Behçet Uz Child Disease and Pediatric Surgery Training and Research Hospital, Department of Pediatrics, İzmir, Turkey; 2 Dr. Behcet Uz Child Disease and Pediatric Surgery Training and Research Hospital, Division of Pediatric Hematology and Oncology, İzmir, Turkey; 3 Ege University Faculty of Medicine, Department of Radiology, İzmir, Turkey; 4 Ege University Faculty of Medicine, Division of Pediatric Hematology and Oncology, İzmir, Turkey

**Keywords:** Acute lymphoblastic leukemia, Iron overload, Complications, Pediatric leukemia

## Abstract

**Objective:**

There are a limited number of studies evaluating iron overload in childhood leukemia by magnetic resonance imaging (MRI). The aim of this study was to determine liver iron content (LIC) by MRI in children with acute lymphoblastic leukemia (ALL) who had completed treatment and to compare those values with serum iron parameters.

**Materials and Methods:**

A total of 30 patients between the ages of 7 and 18 who had completed ALL treatment were included in the study. Serum iron parameters (serum iron, serum ferritin [SF], and total iron-binding capacity) and liver function tests were studied. R2 MRI was performed for determining LIC.

**Results:**

Normal LIC was detected in 22 (63.4%) of the cases. Seven (23.3%) had mild and 1 (3.3%) had moderate liver iron deposition. In contrast, severe iron overload was not detected in any of the cases. LIC levels were correlated with the numbers of packed red blood cell (pRBC) transfusions (r=0.637, p<0.001), pRBC transfusion volume (r=0.449, p<0.013), SF levels (r=0.561, p=0.001), and transferrin saturation (r=0.353, p=0.044). In addition, a positive correlation was found between the number of pRBC transfusions and SF levels (r=0.595, p<0.001).

**Conclusion:**

We showed that the frequency of liver iron deposition was low and clinically less significant after the end of treatment in childhood ALL patients. LIC was demonstrated to be related to SF and transfusion history. These findings support that SF and transfusion history may be used as references for monitoring iron accumulation or identifying cases for further examinations such as MRI.

## Abstract (tr)

**Amaç:**

Çocukluk çağı lösemisinde aşırı demir yükünü manyetik rezonans görüntüleme (MRG) ile değerlendiren sınırlı sayıda çalışma vardır. Bu çalışmanın amacı, akut lenfoblastik lösemisi (ALL) olan ve tedavisini tamamlamış olan çocuklarda R2 MRI yöntemiyle karaciğer demir içeriğini (LIC) belirlemek ve serum demir parametreleriyle karşılaştırmaktır.

**Gereç ve Yöntemler:**

Çalışmaya ALL tedavisini tamamlayan 7-18 yaşları arasında toplam 30 hasta alındı. Serum demir parametreleri (serum demir, serum ferritin [SF] ve toplam demir bağlama kapasitesi), karaciğer fonksiyon testleri çalışıldı. LIC’yi belirlemek için R2 MRG yöntemi kullanıldı.

**Bulgular:**

Olguların 22’sinde (%63,4) normal LIC saptandı. Yedi (%23,3) olguda hafif ve 1 (%3,3) olguda orta derecede karaciğerde demir birikimi mevcuttu. Buna karşılık, hiçbir olguda ciddi derecede demir birikimi tespit edilmedi. LIC seviyeleri, pRBC transfüzyonu sayısı (r=0,637, p<0,001), pRBC transfüzyon miktarı (r=0,449, p<0,013), SF seviyeleri (r=0,561, p=0,001) ve transferrin (r=0,353, p=0,044) ile korele saptandı. Ek olarak, pRBC transfüzyonlarının sayısı ile SF seviyeleri arasında pozitif korelasyon bulundu (r=0,595, p<0,001).

**Sonuç:**

Çocukluk çağı ALL hastalarında tedavi bitiminden sonra karaciğer demir birikiminin sıklığının düşük ve klinik olarak daha az önemli olduğunu gösterdik. Ayrıca, LIC’nin SF ve transfüzyon öyküsü ile ilişkili olduğunu gösterdik. Bu bulgular, SF ve transfüzyon geçmişinin demir birikimini izlemek veya MRG gibi ileri tetkikin gerekli olduğu olguları belirlemek açısından referans olarak kullanılabileceğini desteklemektedir.

## Introduction

Acute lymphoblastic leukemia (ALL) is the most common malignant neoplasm in children and adolescents [1]. Patients require frequent packed red blood cell (pRBC) transfusions, a vital supportive therapy that improves the anemic condition, due to intensive chemotherapy to achieve remission during the induction phase [2,3]. One milliliter of RBCs contains 1.16 mg of iron, and therefore 1 unit of pRBC is estimated to contain about 200-250 mg of iron [2]. However, there is no physiological mechanism involved in the excretion of accumulated iron in the body, and iron overload becomes evident in patients transfused with over 10-20 units of pRBCs [2].

Transfusional iron first accumulates in the bone marrow and reticuloendothelial system (RES) macrophages [4]. When the storage capacity of the RES is exceeded, iron is transferred from macrophages to plasma transferrin. If the iron-binding capacity of transferrin reaches saturation, harmful iron species, such as non-transferrin bound iron (NTBI) and labile plasma iron (LPI), appear in the plasma, resulting in deposition of excess iron within mainly the liver, myocardium, and endocrine organs, which in turn provokes the formation of free radicals, leading to tissue and organ damage [4,5]. There are a limited number of methods that can indicate the amount of iron accumulated in the body that leads to organ damage. Although the determination of iron accumulation by liver biopsy is the most reliable method, it is generally avoided because it is an invasive procedure [6]. In addition, although the serum ferritin level is suggested to be reliable in monitoring iron accumulation in the body, it may be affected by infection or inflammatory conditions and that may lead to misinterpretation [7]. In recent years, magnetic resonance imaging (MRI) has replaced biopsy in the evaluation of body iron accumulation because it is a non-invasive, easily accessible, and reliable method, and it shows the characteristics of the whole tissue iron [8,9]. The principle of this method is based on the paramagnetic properties of iron. Liver iron content (LIC) is indirectly calculated according to the severity of the variability in the magnetic resonance signal caused by iron accumulation in the organ involved. In the literature, there are many studies using different MRI methods for demonstrating liver iron deposition in thalassemia, sickle cell anemia, and myelodysplastic syndrome in children and adults, and in acute leukemia in adults [10,11,12,13,14,15].

There are some studies evaluating iron overload by MRI in children with hematologic malignancies, solid tumors, and hematopoietic stem cell transplantation (HSCT) [16,17,18,19,20,21,22]. In one these studies, liver iron accumulation was determined by the liver/muscle ratio signal intensity (L/M SI) method with lower sensitivity [17], while in other studies, acute leukemias (ALL, acute myeloid leukemia [AML]) and solid tumor cases were evaluated together [16,21]. In a study in which only ALL cases were evaluated, the patients were selected for determination of iron deposition by MRI according to transfusion frequency and/or ferritin level, causing a decrease in the number of cases evaluated (22 of 79 patients included in the study) [18]. Moreover, in some studies, subjects with serum ferritin levels above 1.000 ng/mL were included in the evaluation of iron overload by MRI [19,20]. The aim of the present study was to determine the LIC in children with ALL who had completed treatment by the R2 MRI method and to compare those values with serum iron parameters. This study differs from previous studies in that i) only ALL cases were included, ii) all children had completed treatment, and iii) the R2 MRI method was applied in all cases.

## Materials and Methods

### Patient Selection

A total of 32 patients between the ages of 7 and 18 who had completed ALL treatment between January 2012 and December 2013 were included in the study. Two patients were excluded from the study because they did not want to be included or due to technical errors in liver MRI. As a result, 11 females (median age: 8.2; range: 7.1-17.2 years) and 19 males (median age: 7.5; range: 7.1-15.7 years) were included in the study. According to the ALL BFM 2009 protocol, 13 patients were in the standard-risk (SR), 15 were in the intermediate-risk (IR), and 2 were in the high-risk (HR) group. Patients younger than 7 years of age, those who were noncompliant with the commands during MRI, and those with mental retardation, fear of tight spaces (claustrophobia), or hepatitis were excluded from the study. This study was approved by the Ethics Committee of Dr. Behçet Uz Children’s Hospital in İzmir, Turkey, in accordance with the Declaration of Helsinki (date/number: 12.12.2011/72). All of the children and/or their parents gave written informed consent before the study.

The numbers of pRBC transfusions and the transfusion volumes received during the treatment period were recorded. At the end of treatment, serum iron parameters (serum iron, serum ferritin [SF], and total iron-binding capacity [TIBC]), liver function tests (alanine transaminase [ALT], aspartate transaminase [AST], albumin, total bilirubin, direct bilirubin), prothrombin time (PT), and partial thromboplastin time (APTT) were studied. SF levels were determined by a chemiluminescent microparticle immunoassay (Abbott, Architect i2000) and serum iron and TIBC were determined with an Abbott Architect c1600 autoanalyzer. Transferrin saturation was calculated by dividing serum iron into TIBC and expressed as a percentage. At the same time as the evaluation of serum iron profile, R2 MRI was performed in the Ege University MRI Unit in İzmir for determining LIC. MRI evaluations were performed 1-5 months (median: 3 months) after the end of treatment. Serum iron, SF, and TIBC levels and transferrin saturation calculations were repeated after an interval of 9-13 months (median: 11 months) for patients with high SF levels (>500 ng/mL) detected in the first evaluation.

### Magnetic Resonance Imaging and Liver Iron Content Analysis

All MRI examinations of the liver were performed with a 1.5-T scanner (Symphony, Siemens, Erlangen, Germany) using body and head coils, respectively. Liver-R2 values of the ALL patients were measured. To calculate liver-R2, liver images were collected from the same respiratory-triggered slices of axial multi-echo SE sequences (TR: 300 ms; TE: 3.8, 6, 9, 12, 15, and 18 ms; slice thickness: 10 mm; matrix size: 80x256; FOV: 42 cm). The scan durations were 84 s each.

MRI images of the liver were initially examined in order to exclude the presence of any mass lesions. Afterwards, the echo images of the pituitary gland and liver were exported from the scanner in DICOM-3 format and imported to dedicated software that cooperates with MATLAB (MathWorks, Natick, MA, USA). A mono-exponential function of the formula S(TE) = So x exp(-TE x R2) + C was used as a representative of the signal decay with echo time (TE), where S(TE) is the signal at TE. Therefore, the signal amplitude at TE is 0, and C is the constant offset parameter added to compensate for background noise bias [23,24]. To assess hepatic iron accumulation, the largest region of interest (ROI) was drawn around the entire liver boundary of the first echo, excluding hilar vessels, the vena cava inferior, and motion artifacts. The ROI was copied to all images of the corresponding echoes. R2 evaluation of the liver was performed with the same analytic tool using the same MATLAB equations. The corresponding R2 maps of the liver were derived. The mean and median R2 values and standard deviations were calculated. The mean R2 value within the liver was then used to yield a mean LIC using a calibration curve that had been determined through the measurement of the liver R2 and needle-biopsy LIC [8]. Analysis of all MRI examinations was performed by the same experienced radiologist.

The LIC values obtained for patients were classified according to the levels defined by Olivieri and Brittenham [4] in thalassemia patients. Accordingly, LIC was evaluated as normal for values of <3.2 mg/g dry weight (d.w.), as mild iron overload for values of 3.2-7.0 mg/g d.w., as moderate iron overload for values of 7.0-15.0 mg/g d.w., and as severe iron overload for values of >15 mg/g d.w.

### Statistical Analysis

Statistical analyses of the data were conducted with SPSS 20.0 (IBM Corp., Armonk, NY, USA). The distribution of the data was evaluated with the Shapiro-Wilk test. For numerical comparisons, independent sample t-tests or Mann-Whitney U tests were used according to the normal distribution of the measured parameters. In the comparison of two dependent groups (alterations in iron parameters during follow-up), paired t-tests or Wilcoxon rank tests were performed according to the normal distribution of the parameters. Spearman’s rho correlation was used to identify the associations between variables. Categorical data were expressed as frequency (%), while numerical data were expressed as median (25^th^-75^th^ percentiles) or mean ± standard deviation. In all statistical tests, p<0.05 was considered significant.

## Results

Thirty patients (11 females, 19 males; median age: 7.8 years) were included in the study. Thirteen patients were in the SR group, 15 were in the IR group, and 2 were in the HR group. The median transfusion number was 12 (25^th^-75^th^ percentiles: 8-16) and the median SF value was 454.5 (25^th^-75^th^ percentiles: 120-894). In 6 patients, the SF values were between 500 and 1.000 ng/mL, and 4 had SF levels above 1.000 ng/mL. ALT, AST, total bilirubin, direct bilirubin, albumin, PT, APTT, and INR values were within the normal ranges in all patients. The median LIC was 2.77 mg/g d.w. (25^th^-75^th^ percentiles: 1.28-3.43) (Table 1).

Normal LIC (<3.2 mg/g d.w.) was detected in 22 (63.4%) of the patients. Seven (23.3%) had mild (3.2-7.0 mg/g d.w.) and 1 (3.3%) had moderate (8.47 mg/g d.w.) liver iron deposition. Severe iron overload was not detected in any of the cases. The characteristics of the patients with mild to moderate liver iron overload are summarized in Table 2.

**Figure 1:**
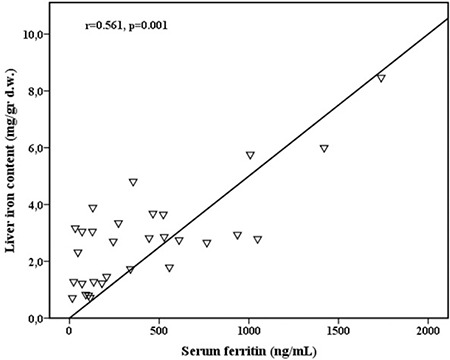
Demonstration of the correlation between LIC and serum ferritin levels.

When the patients were divided into two groups according to LIC levels, the numbers of pRBC transfusions, total pRBC transfusion volumes, and SF levels were significantly higher in patients with LIC of >3.2 mg/g d.w. (p<0.05) (Table 3). In addition, LIC levels were positively correlated with the number of pRBC transfusions (r=0.637, p<0.001), RBC transfusion volume (r=0.449, p<0.013), SF levels (r=0.561, p=0.001) (Figure 1), and transferrin saturation (r=0.353, p=0.044) and negatively correlated with TIBC (r=-0.439, p<0.001) (Table 4). In addition, a positive correlation was found between the number of pRBC transfusions and SF levels (r=0.595, p<0.001).

Serum iron, transferrin saturation, TIBC, and SF levels were measured again after 9-13 months (median: 11 months) for patients with high SF levels (>500 ng/mL) detected at the first evaluation. The decreases in SF levels from 773 ng/mL (508-1141) to 460 ng/mL (265-582) (p=0.005), in serum iron levels from 122 µg/dL (84-142) to 71.5 µg/dL (57.7-91.1) (p=0.005), and in transferrin saturation from 36.5% (29%-50%) to 29% (19%-34%) (p=0.026) and the increase in serum TIBC from 330.1±35 µg/dL to 437.2±163.9 µg/dL (p=0.047) were statistically significant.

## Discussion

Anemia occurs frequently in childhood ALL cases due to the intensive chemotherapy given in the first 6 months of treatment. Therefore, iron accumulation may occur in this period due to intensive transfusions. Limited data are available for assessing iron accumulation due to transfusion in childhood acute leukemia. Although liver biopsy is considered to be the gold standard for measuring iron deposition, it has become a less commonly used method because of its invasive nature. In the current study, MRI, which is an indirect measurement method but strongly correlated with liver biopsy findings, was used for LIC determination [8,9,11,16,17,25,26]. Iron accumulation was not detected in most of our patients (63.4%), while mild iron deposition was found in 23.3% of patients and moderate iron deposition was found in 1 (3.3%). No severe iron accumulation was detected in any of the patients. In adult studies in which liver iron deposition was evaluated by MRI, moderate iron overload was reported in 50% of 88 patients with myelodysplastic syndrome and AML undergoing allogenic HSCT by Wermke et al. [14], moderate iron accumulation was identified in 22.2% of 27 patients with acute leukemia (ALL, AML) by Yassin et al. [15], and hepatic iron overload over 5 mg/g d.w. was found in 48% of 42 patients undergoing myeloablative HSCT by Armand et al. [10]. The reason for the higher incidence of moderate iron accumulation in adult cases than in our cohort is thought to be related to the fact that most of the cases in adult studies are more severe (MDS, myeloablative stem cell transplantation, AML, etc.) and require more frequent transfusion. There are some studies in the literature evaluating liver iron deposition in childhood cancer by MRI. Vag et al. [17] evaluated liver iron accumulation during treatment in 15 children with acute leukemia (9 ALL, 6 AML) by measuring the L/M SI ratio (liver/vertebral muscle signal intensity) by gradient-recalled echo method. They showed a significant decrease in signal intensity in the liver due to transfusion-dependent iron overload; however, they could not report an estimation for LIC due to the selected method’s limitations [17]. Ruccione et al. [16] evaluated liver iron deposition by MRI in 75 children and young adults (age range: 8-25.6 years) with acute leukemia (ALL/AML; n=33) and solid tumors (n=42) and high LIC levels were reported in 49.3% of the patients. In the same study, co-evaluation of acute leukemia (ALL/AML) and solid tumors prevented inferences about iron accumulation in acute leukemia. Unal et al. [18] reported 50% liver and 27% cardiac iron deposition in 36 ALL patients (28 on treatment, 8 off treatment) by T2* MRI and, in addition, liver iron accumulation was demonstrated in two cases among 8 patients whose treatment had been completed. In the same study, it was concluded that the number of erythrocyte transfusions is more reliable than SF levels in screening for iron accumulation. In two different studies by Olcay et al. [19,20], liver iron deposition was shown in 75% and 40% of patients with acute leukemia (AML and ALL) with SF levels above 1000 ng/mL. It was suggested that since iron accumulation can be detected even when the erythrocyte transfusions are not intense, this accumulation may be related to the intensity of chemotherapy treatment [19]. Moreover, they reported that no association was found between serum levels of NTBI, which is thought to cause tissue damage, and oxidative stress markers (lipid hydroperoxide products and protein carboxyl groups) and liver iron deposition [20]. In another study, de Ville de Goyet et al. [21] reported that liver iron accumulation was found to be 66% in the first year and 82% in the second year in pediatric patients with hematologic malignancy or solid tumor. They also showed a close relationship between iron accumulation and the intensity of treatment received and number of transfusions. In a study conducted by Schempp et al. [22], it was shown that the frequency of iron overload in patients with a history of allogenic HSCT (25.9%) was significantly higher than in those treated without HSCT (3.7%) and with autologous HSCT (0%). In our study, iron accumulation was found in 26.6% of cases (7 mild, 1 moderate), which is comparable with previous studies on childhood malignancies. All of these findings suggest that liver iron accumulation can be detected during treatment or may continue after cessation of treatment.

Unlike thalassemia, it is not clear how to interpret LIC values in acute leukemia patients who require RBC transfusions during the induction period of treatment. However, it is recommended that 7 mg/g d.w. be considered as a cut-off in the prediction of clinical complications [4,13,27]. When we evaluated the LIC results of our cohorts according to this cut-off value, we concluded that one patient (3.3%) had iron accumulation with a risk of clinical complications. During the follow-up of that case, however, no impairment in liver function tests was observed.

The primary determinant of liver iron deposition is known to be related to the amount of transfusions administered to the patient. In our study, the number of transfusions and the total transfusion volumes were found to be significantly higher in patients with LIC of  >3.2 mg/g d.w. In addition, in the current study, LIC had a strong correlation with the number of transfusions and with the amounts of the transfusions. A similar positive relationship has been shown in many studies [7,10,13,15,16,18,21]. In addition, in our study, LIC levels were positively correlated with SF and transferrin saturation, while they were negatively correlated with TIBC. Many studies in children and adults with thalassemia, sickle cell anemia, MDS, and acute leukemia have reported strong correlations between LIC and SF levels [4,10,13,14,17,23,28]. These findings suggest that although the SF level is severely affected by inflammation and infection, it is still a good indicator for monitoring iron deposition as it is easily accessible, relatively inexpensive, and well standardized. Furthermore, efforts have been made to determine the cut-off values for SF level and transfusion numbers to estimate liver iron accumulation. In some studies, the transfusion of over 20 units of pRBCs has been suggested to be a significant indicator of severe iron accumulation [29,30]. Despite that, it was reported that significant iron accumulation can be detected in cases of hematologic malignancies (unlike thalassemia and sickle cell anemia) even when the number of transfusions is below 10 [31]. In addition, it has been stated that SF levels above 1000 ng/mL are good indicators of iron accumulation and can be used in clinical follow-up [29,32,33]. However, there are also studies suggesting that the cut-off value of SF level should be higher to predict remarkable iron accumulation with high sensitivity [10,14]. In one study, it was suggested that the risk of significant liver iron accumulation was low in patients who received fewer than 15 units of transfusion and whose ferritin level was found to be below 1000 ng/mL [10]. In contrast, iron accumulation was also reported in patients with SF levels below 1000 ng/mL [18,19]. Iron accumulation in these cases has been suggested to be associated with increased numbers of pRBC transfusions [18] and active leukemia with its own effects or with anti-leukemic treatment [19]. Consistently, we demonstrated mild liver iron deposition in 3 patients (numbers of pRBC transfusions above 10) with SF levels below 500 ng/mL and in 1 patient (number of pRBC transfusions below 10) with SF level between 500 and 1000 ng/mL. These findings suggest that iron accumulation can be detected even when the SF level is lower than the estimated threshold of 1000 ng/mL. Moreover, in the current study, when the pRBC transfusion cut-off was assigned as 15 (sensitivity: 55.5%, specificity: 85.8%) and the ferritin level cut-off was assigned as 1000 ng/mL (sensitivity: 75%, specificity: 80.8%), a significant difference was found in terms of LIC. Although we demonstrated that LIC levels were positively correlated with the number pRBC transfusions and SF levels, it may be less misleading to estimate iron accumulation accurately by determining a clear cut-off for SF or the numbers of transfused pRBC units. Further studies with large case series are needed, taking into account the effect of the malignancy itself and the effect of chemotherapeutic agents on facilitating iron deposition.

Since ferritin levels correlate with LIC, it is widely believed that ferritin levels can be used in follow-up. In a study conducted by Halonen et al. [7] including 30 patients with ALL, they performed liver biopsy and measured serum iron parameters to determine liver iron deposition at the end of treatment, and then serum iron parameters were re-evaluated 1-3 years after the end of treatment. They reported good correlations between LIC, SF, transferrin saturation, and transfusion numbers. In the same study, it was reported that there was a statistically significant decrease in SF, serum iron, and transferrin saturation values measured during follow-up. Similarly, in our study, control serum iron and ferritin levels and transferrin saturation were significantly decreased in patients who had high ferritin levels measured in the initial evaluation. Although excess iron accumulation in the body cannot be eliminated by any mechanism, it can be predicted that it may decrease due to the increasing need in childhood growth and development periods [18,34].

Some limitations of the present study have to be acknowledged. The first limitation is that the small number of participants in the subgroup analyses might have negatively affected the results. In addition, the low number of subgroups prevented us from performing some evaluations related to ALL risk groups. For example, subgroup comparisons or correlation analyses could not be performed because most patients were in the SR and IR groups and there were only 2 in the HR group. Selection bias cannot be ruled out in this cohort because patients under 7 years of age, who cannot tolerate MRI, were not included in the study. Moreover, serum parameters were measured and MRI evaluations of patients were performed 1-5 months (median: 3 months) after cessation of treatment. Although there is no excretory mechanism for iron in the human body and so the only mechanism for its clearance is through epithelial sloughing, bleeding, or iron utilization by erythropoietic tissue [35], the period between the end of treatment and MRI assessment might cause slight clearance of liver iron and lower LIC measurements. Finally, various hemochromatosis gene polymorphisms that lead to mild and moderate iron deposition and mild clinical phenotypes have been described [36]. Our patients were not evaluated in this respect. However, a significant decrease in SF levels in the follow-up period may be considered as evidence that this was not the case.

## Conclusion

In this study, we have shown that the frequency of liver iron deposition was low and clinically less significant after the end of treatment in childhood ALL patients of the SR and IR subgroups. LIC was demonstrated to be related to SF and transfusion history. These findings support that SF and transfusion history may be used as references for monitoring iron accumulation or identifying cases for further examination such as MRI. However, long-term longitudinal studies with larger case series including HR patients receiving treatment are needed to establish more accurate cut-off values to confirm LIC estimation or to verify our findings.

## Figures and Tables

**Table 1:**
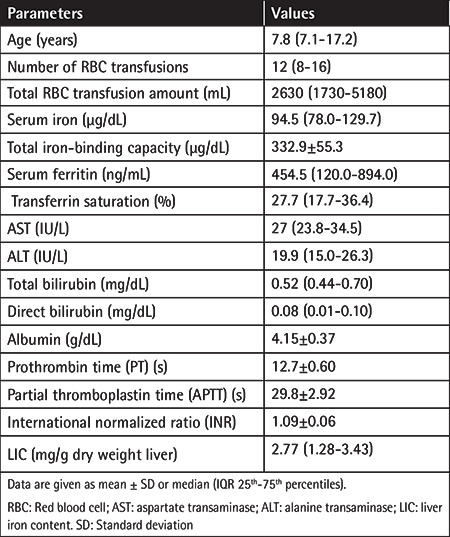
Evaluation of age, transfusion history, serum iron parameters, and LIC data of the patients included in the study.

**Table 2:**
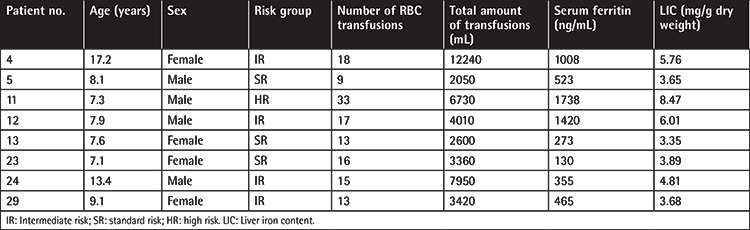
Clinical and laboratory characteristics of patients with mild and moderate iron deposition.

**Table 3:**
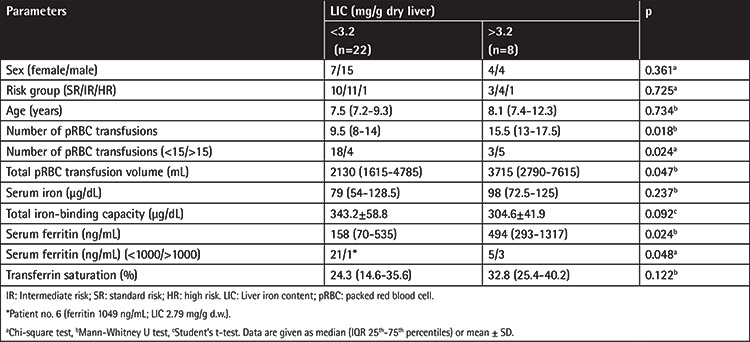
Comparison of serum iron parameters and transfusion data according to liver iron accumulation status.

**Table 4:**
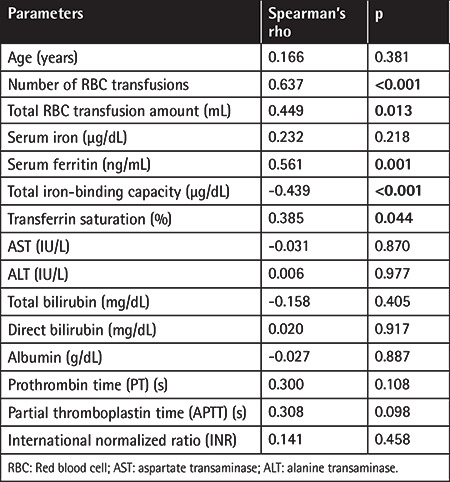
Evaluation of the relationship between LIC and age, transfusion number, iron parameters, and liver function tests.
